# Caffeine Restores Neuronal Damage and Inflammatory Response in a Model of Intraventricular Hemorrhage of the Preterm Newborn

**DOI:** 10.3389/fcell.2022.908045

**Published:** 2022-08-12

**Authors:** Pilar Alves-Martinez, Isabel Atienza-Navarro, Maria Vargas-Soria, Maria Jose Carranza-Naval, Carmen Infante-Garcia, Isabel Benavente-Fernandez, Angel Del Marco, Simon Lubian-Lopez, Monica Garcia-Alloza

**Affiliations:** ^1^ Division of Physiology, School of Medicine, Universidad de Cadiz, Cadiz, Spain; ^2^ Biomedical Research and Innovation Institute of Cádiz Cadiz (INiBICA) Research Unit, Puerta del Mar University Hospital University of Cadiz, Cadiz, Spain; ^3^ Salus-Infirmorum, University of Cadiz, Cadiz, Spain; ^4^ Area of Pediatrics, Department of Child and Mother Health and Radiology, Medical School, University of Cadiz, Cadiz, Spain; ^5^ Section of Neonatology, Division of Pediatrics, Hospital Universitario Puerta del Mar, Cadiz, Spain

**Keywords:** preterm infant, germinal matrix-intraventricular hemorrhage, caffeine, atrophy, microglia, hemorrhage, cognition

## Abstract

Germinal matrix-intraventricular hemorrhage (GM-IVH) is the most frequent intracranial hemorrhage in the preterm infant (PT). Long-term GM-IVH-associated sequelae include cerebral palsy, sensory and motor impairment, learning disabilities, or neuropsychiatric disorders. The societal and health burden associated with GM-IVH is worsened by the fact that there is no successful treatment to limit or reduce brain damage and neurodevelopment disabilities. Caffeine (Caf) is a methylxanthine that binds to adenosine receptors, regularly used to treat the apnea of prematurity. While previous studies support the beneficial effects at the brain level of Caf in PT, there are no studies that specifically focus on the role of Caf in GM-IVH. Therefore, to further understand the role of Caf in GM-IVH, we have analyzed two doses of Caf (10 and 20 mg/kg) in a murine model of the disease. We have analyzed the short (P14) and long (P70) effects of the treatment on brain atrophy and neuron wellbeing, including density, curvature, and phospho-tau/total tau ratio. We have analyzed proliferation and neurogenesis, as well as microglia and hemorrhage burdens. We have also assessed the long-term effects of Caf treatment at cognitive level. To induce GM-IVH, we have administered intraventricular collagenase to P7 CD1 mice and have analyzed these animals in the short (P14) and long (P70) term. Caf showed a general neuroprotective effect in our model of GM-IVH of the PT. In our study, Caf administration diminishes brain atrophy and ventricle enlargement. Likewise, Caf limits neuronal damage, including neurite curvature and tau phosphorylation. It also contributes to maintaining neurogenesis in the subventricular zone, a neurogenic niche that is severely affected after GM-IVH. Furthermore, Caf ameliorates small vessel bleeding and inflammation in both the cortex and the subventricular zone. Observed mitigation of brain pathological features commonly associated with GM-IVH also results in a significant improvement of learning and memory abilities in the long term. Altogether, our data support the promising effects of Caf to reduce central nervous system complications associated with GM-IVH.

## 1 Introduction

Very low birth weight infants (VLBWIs) are those preterm infants (PT) born at or under 32 weeks of gestational age. There are more than 15 million premature births worldwide every year, and preterm birth is the leading cause of death in children (Walani, 2020). Although in recent years survival rates of PT have increased due to advances in neonatal intensive care and perinatal medicine, this is also accompanied by an increase in morbidities associated with prematurity (for review (Atienza-Navarro et al., 2020)). Therefore, this preterm population is at high risk for complications such as germinal matrix-intraventricular hemorrhage (GM-IVH), which is not only one of the most common pathologies associated with prematurity but also the most frequent intracranial hemorrhage in the PT (da Silva et al., 2018). Although incidences may vary depending on the studies and populations assessed, approximately 20%–30% of all PT may suffer GM-IVH of any grade, and severe GM-IVH may affect between 6% and 16% of all PT (Stoll et al., 2010; Bolisetty et al., 2014; Radic et al., 2015a; Khanafer-Larocque et al., 2019). Moreover, severe GM-IVH is the leading cause of death in PT with a gestational age at birth under 28 weeks (Harkin et al., 2019).

The germinal matrix (GM) is a highly vascularized region that fully covers the ventricle. It is also a source of neuronal and glial cells in the immature brain that will maturate and migrate to their final destinations along development (Segado-Arenas et al., 2017; Cerisola et al., 2019). The immaturity of the central nervous system (CNS) of the PT, its hemodynamic instability (Szpecht et al., 2016), and the difficulty to autoregulate cerebral blood flow (Rellan Rodríguez et al., 2008) make the fragile GM vasculature prone to bleed (Atienza-Navarro et al., 2020; Lampe et al., 2020). Since GM commences to involute by 28 gestational weeks until it disappears in full-term kids (Mukerji et al., 2015; Segado-Arenas et al., 2017), GM-IVH affects almost exclusively VLBWIs (da Silva et al., 2018). GM-IVH–associated morbidities (Christian et al., 2016) include cerebral palsy, sensory and motor impairment, and learning disabilities among others (for review (Atienza-Navarro et al., 2020)). Although the harmful effects of severe GM-IVH seem unquestionable (Bolisetty et al., 2014; Mukerji et al., 2015), previous studies have also reported that any degree of GM-IVH may predispose to suffer these complications (Radic et al., 2015b; Iyer et al., 2015). GM-IVH patients also have a higher risk to develop psychiatric disorders such as autism spectrum disorders, anxiety, depression, or altered social behaviors (You et al., 2019; Chung et al., 2020).

The societal and health burden associated with prematurity and GM-IVH concretely, is worsened by the fact that there is no successful treatment to limit or reduce brain damage and neurodevelopment disabilities. Different options, such as prophylactic vitamin K, coagulating factors, angiogenic inhibitors, COX-2 inhibitor celecoxib, or endothelial growth factor R2 inhibitors, might have beneficial effects in the GM-IVH management by stabilizing the GM at different levels, but more studies are required before use in clinical care (for review (Deger et al., 2021)). Other approaches have focused on caffeine (Caf), regularly used to treat the apnea of prematurity (Long et al., 2021). Caf has antioxidant, neuroprotective, and anti-inflammatory properties, and studies in newborn and young animals have shown its capacity to reduce brain hypoxic damage (Yang et al., 2021). Similarly, studies in patients have reported that Caf treatment in VLBWI with apnea of prematurity may improve neurodevelopmental outcomes (Schmidt et al., 2007; Maitre et al., 2015). Nevertheless, to our knowledge, the direct effect of Caf on GM-IVH remains undetermined, and there is only one study assessing the role of Caf in GM-IVH (MRI and Neurodevelopment in PT Following Administration of High-Dose of Caf. ClinicalTrials.gov. Identifier: NCT00809055) reporting beneficial long-term effects of Caf treatment (Mürner-Lavanchy et al., 2018). Therefore, we have used an animal model of GM-IVH to specifically assess the effect of two doses of Caf (10 and 20 mg/kg/day) on brain pathology and cognitive performance. We have observed that both in the short term (P14) and in the long term (P70), Caf reduces brain atrophy. Also, mature neurons population and neuron curvature are significantly improved. Likewise, reduced proliferation and neurogenesis in the subventricular zone (SVZ) are ameliorated by Caf treatment. Furthermore, cortical and SVZ bleeding and inflammation are reduced, altogether resulting in better learning and memory capabilities in the long term, in line with previous observations in other models of perinatal insult (Endesfelder et al., 2017a; Yang et al., 2022). Ultimately, our results may help better understand the pathological features associated with GM-IVH in the PT and to elucidate the beneficial effects associated with Caf treatment.

## 2 Methods

### 2.1 Animals and Treatments

For the experiment, 7-day-old (P7) CD1 mice received 0.3 IU of collagenase (Col) (purified collagenase VII, batch SLBG8830V, Sigma-Aldrich, St Louis, MO, United States) in 1 µl of TESCA (TES buffer 50mM and calcium chloride anhydrous 0.36 mM) administered intracerebroventricularly, as previously described (Segado-Arenas et al., 2017). Briefly, mice were anesthetized with isoflurane, immobilized in a stereotactic frame (David-Kopf, CA, United States), and Col was administered at 0.1 μl/min for 10 min using a 10-μl Hamilton syringe (Hamilton Company, United States) in the right ventricle (AP −3 mm, ML −1 mm, and DV +4 mm, using Bregma as reference). The needle was left in the lesion site for 5 min after completing the injection. Sham animals underwent the same surgical procedures but received 1 µl of TESCA. Animals were allowed to recover and were returned to the home cages with their mothers. A naïve group of animals did not undergo any surgical procedures. Animals were treated with Caf (Sigma-Aldrich, St Louis, MO, United States) (10 or 20 mg/kg/day) intraperitoneally (i.p.) (Kaindl et al., 2008; Fan et al., 2011) for 3 consecutive days, commencing immediately after the lesions (P7–P9), and untreated animals received filtered phosphate buffer (PBS) instead. A first set of animals were analyzed at P14, and a second set of mice were analyzed at P70 to assess the effects of Caf in the short and long terms.

Body weight was measured before the lesions and before sacrifice at P14 and P70. All experimental procedures were approved by the Animal Care and Use Committee of the University of Cadiz, in accordance with the guidelines for care and use of experimental animals (European Commission Directive 2010/63/UE and Spanish Royal Decree 53/2013).

### 2.2 Morris Water Maze (MWM)

The MWM test commenced at P56, and it was performed as previously described (Segado-Arenas et al., 2017). Briefly, the pool consisted of a round tank 90 cm in diameter surrounded by external clues, with a platform hidden under the water. Water temperature was 21 ± 2 °C. The acquisition phase included four trials per day for 4 consecutive days. The time limit was 60 s/trial, with a 10-min inter-trial interval. If the animal did not find the platform, it was placed on the platform for 10 s. The retention phase was performed 24 and 72 h after the completion of the acquisition phase and consisted in a single trial with the platform removed. Time required to locate the platform in the acquisition phase, the number of entrances in quadrant 2, where the platform was located, during the retention phase, and swimming speed were analyzed by Smart software (Panlab, Barcelona, Spain).

### 2.3 Actimetry and the New Object Discrimination (NOD) Test

Spontaneous motor activity was analyzed the day after completing the MWM test by measuring the walking distance for 30 min in a rectangular box (44 cm long × 22 cm width × 40 cm high). The NOD test was commenced 24 hours later to analyze episodic memory. Animals were exposed to two objects for 5 min for habituation purposes, which were not used again during the test. The next day each mouse received two sample trials and a test trial. On the first sample trial, mice were allowed to explore for 5 min, with four copies of a novel object (navy balls) arranged in a triangle-shaped spatial configuration. After a 30-min delay, mice received a second sample trial with four novel objects (red cones) arranged in a quadratic-shaped spatial configuration, for 5 min. After 30 min, the mice received a test trial with two copies of the object from sample trial 2 (“recent” objects) placed at the same position, and two copies of the object from sample trial 1 (“familiar” objects) with one of them being placed at the same position (“old non displaced” object) and the other in a new position (“familiar displaced” object). An integrated episodic memory for “what,” “where,” and “when” paradigms were analyzed are shown, as previously described (Dere et al., 2005). “What” was defined as the difference in time exploring familiar and recent objects, “where” was defined as the difference in time exploring displaced and nondisplaced objects, and “when” was defined as the difference between time exploring familiar nondisplaced and recent nondisplaced objects.

### 2.4 Rotarod

Motor skills and motor coordination were also evaluated by the rotarod (Panlab Harvard Apparatus, Barcelona, Spain), as described (Segado-Arenas et al., 2017). The animal was placed for 3 min at 4 rpm for habituation purposes on the horizontal rod (3 cm in diameter and 5.7 cm wide). During the test, the speed was increased from 4 to 40 rpm within 1 min. The time spent on the rod and the velocity when the animal fell were recorded.

### 2.5 Tissue Processing

Animals were sacrificed by an overdose of pentobarbital (Richter Pharma, Wels, Austria) (120 mg/kg) administered i.p. Brains were harvested and weighed. While the ventricle provides complete access to the brain, our previous characterization of the model revealed that the right hemisphere was more severely affected after Col administration in the right ventricle (Segado-Arenas et al., 2017). Therefore, all of our *postmortem* analyses were performed on the brain structures of the right hemisphere (the cortex, SVZ, and hippocampus), as described (Segado-Arenas et al., 2017). The brains from half of the animals were fixed in 4% paraformaldehyde (PFA) for 3 weeks and cryoprotected in 30% sucrose, and serial coronal sections of 30 μm were cut on a cryostat (Thermo-Scientific, Microm HM 525, Germany). The sections were stored at −20°C in PBS and glycerol (1:1) until ipsilateral hemispheres were used. The brains from the remaining animals were dissected, and the ipsilateral cortex, striatum, and hippocampus were immediately frozen at −80°C for biochemical studies.

### 2.6 Cresyl Violet Staining

Six coronal sections of 30 μm at 1.5, 0.5, −0.5, −1.5, −2.5, and −3.5 mm from Bregma were selected (Segado-Arenas et al., 2017; Infante-Garcia et al., 2018). Sections were mounted on Superfrost™ slides (Thermo-Fisher Scientific, Waltham, MA, United States), dehydrated in 70% ethanol for 15 min, and incubated in 0.5% cresyl violet solution for 10 min. Tissues were fixed in 0.25% acetic acid in ethanol for 7 min, followed by ethanol for 2 min and xylol for 2 more minutes. Sections were mounted with DPX medium (Sigma Aldrich, St Louis, MO, United States). Images were photographed with a ×4 objective on an Olympus B×60 fluorescence microscope (Olympus, Tokyo, Japan) coupled to an Olympus DP71 camera, by MMIcellTools software (Olympus, Hamburg, Germany). Adobe Photoshop Elements software was used to photomerge all images and build complete sections. ImageJ was used to measure the total hemisection size, cortex, hippocampus, and ventricle areas. Quantifications were carried out in blind experiments for the person performing the measurements.

### 2.7 Prussian Blue Staining

Contiguous sections to those used for cresyl violet staining were selected to analyze small vessel bleeding, as described (Desestret et al., 2009) with minor modifications. Sections were dehydrated and incubated in Prussian blue solution (20% HCl and 10% potassium ferrocyanide) for 30 min. They were rinsed with distilled water and rehydrated in PBS for 5 min. Sections were counterstained with neutral red (1% neutral red, 1% acetic acid) for 5 min. Sections were washed and dehydrated for 2 min with increasing concentrations of ethanol (95%, 99% + 1% of acetic acid, and 100%) and xylol. Sections were covered with DPX and photographed with an Olympus DP71 camera attached to an Olympus Bx60 microscope (Olympus, Tokyo, Japan) with a ×6.4 objective. Adobe Photoshop Elements software was used to photomerge all images and build complete sections. ImageJ software was used to quantify the hemorrhage burden (area occupied by hemorrhages) in the cortex and the SVZ.

### 2.8 NeuN/DAPI/Iba-1 Staining

Six coronal sections of 30 µm contiguous to those previously used were selected and incubated overnight at 4 °C with the anti-NeuN antibody (MAB377, Sigma, St. Louis, MO, United States) (1:200) to label mature neurons, and anti-Iba-1 (019-19741, Wako, Osaka, Japan) (1:1,000) as a microglia marker (Infante-Garcia et al., 2017a). Sections were incubated with anti-IgG-mouse Alexa Fluor 488 (1:1,000) (Thermo Fisher Scientific, Waltham, MA, United States) and anti-IgG-goat Alexa Fluor 594 (1:1,000) (Thermo Fisher Scientific, Waltham, MA, United States) for 1 h, followed by counterstaining with DAPI 1 mg/ml (Sigma, St. Louis, MO, United States) (1:3,000) for 10 min. Sections were photographed using an Olympus Bx60 fluorescence microscope (Olympus, Tokyo, Japan) coupled to an Olympus DP71 camera with a ×16 objective.

Twenty ROIs (7,451.062 µm^2^/ROI) were selected in each cortical section, and eight ROIs were also selected in three sections 1 mm apart (1.5 to −0.5 mm from Bregma) which comprised the SVZ. The percentage of NeuN-positive cells (normalized by total cells stained with DAPI) was quantified in the cortex and the SVZ by ImageJ software (Ramos-Rodriguez et al., 2017).

Microglia burden (% covered area by Iba-1 immunostaining) in the cortex and the SVZ was also quantified by analyzing 20 ROIs/section in the cortex and eight ROIs/section in the SVZ by ImageJ software, as described (Infante-Garcia et al., 2016).

### 2.9 SMI-312 Immunostaining

The axonal curvature was analyzed with the anti-neurofilament marker SMI-312 antibody (Infante-Garcia et al., 2017b) in contiguous sections to those previously used. Sections were pretreated with 3% hydrogen peroxide and 0.5% Triton-X for 10 min and blocked with 3% bovine serum albumin (BSA) for 3 h. Thereafter, the sections were incubated with anti-SMI-312 (1:1,000) (837904, BioLegend, San Diego, CA, United States) overnight at 4°C. Anti-IgG-mouse Alexa Fluor 594 was used as a secondary antibody (Thermo Fisher Scientific, Waltham, MA, United States) (1:200). Images were acquired with an Olympus Bx60 fluorescence microscope (Olympus, Tokyo, Japan) coupled to an Olympus DP71 camera with a 40X objective and MMIcellTools software. The axon curvature ratio was calculated by dividing the end-to-end distance of a neurite segment by the total length between the two segment ends. Neurites had at least 20 µm of length (Infante-Garcia et al., 2017b). At least 150 neurites in the cortex and 45 neurites in the SVZ of each animal were analyzed by ImageJ software.

### 2.10 Ki67 and Doublecortin (DCX) Immunostaining

Proliferation and neurogenesis were analyzed in the SVZ. Three sections 1 mm apart (1.5 to −0.5 mm from Bregma) were selected. To analyze proliferation and neurogenesis, the anti-Ki67 antibody (ab15580, Abcam, Amsterdam, Netherlands) (1:200) and anti-DCX antibody (sc-271390, Santa Cruz Biotechnology, Inc., Texas, United States) (1:50) were used as described in Hierro-Bujalance et al. (2020a). Sections were pretreated with citrate formamide (1:1) for 2 h at 65°C. Thereafter, sections were incubated in 2N HCL for 30 min at 37°C and placed in a 0.1M borate buffer at pH 8.5. After blocking with 3% BSA and 0.5% TritonX-100 for 1 h, sections were incubated with primary antibodies overnight at 4 °C. Secondary antibodies anti-IgG-rabbit Alexa Fluor 488 (Thermo Fisher Scientific, Waltham, MA, United States) (1:1,000) and anti-IgG-mouse Alexa Fluor 594 (Thermo Fisher Scientific, Waltham, MA, United States) (1:1,000) were used. Confocal images of 30 µm in depth were acquired with a Z-step size of 2 μm. A ×20 objective on a Zeiss LSM 900 Airyscam 2 confocal microscope (Zeiss, Oberkochen, Germany) was used.

ImageJ software was used to analyze DCX burden (percentage of area covered by DCX^+^ cells), density of Ki67^+^ cells, and overlapping DCX^+^ area/Ki67^+^ cells in the SVZ, as described in Ramos-Rodriguez et al. (2014), considering the SVZ area comprised in the first 100 μm adjacent to the ventricle lumen.

### 2.11 Total-Tau and Phospho-Tau Levels

Total-tau (10736333, Invitrogen, Thermo-Fisher Scientific, Waltham, MA, United States) and phospho-tau levels [pS199] (10272883, Invitrogen, Thermo-Fisher Scientific, Waltham, MA, United States) were measured in the cortex and striatum samples by colorimetric ELISA kits, following the manufacturer’s instructions. Briefly, 10 mg of tissue were homogenized in 50 µl of homogenization buffer (5 M guanidine-HCl diluted in 50 mM Tris) with protease and phosphatase inhibitor cocktail for 20 min on ice. The homogenate was centrifuged at 14,500×g for 5 min at 4 °C, and the supernatant was collected for ELISA assay. Absorbances were measured at 450 nm in a spectrophotometer (MQX200R2, BioTek Instruments, Burlington VT, United States). Phospho-tau/total-tau ratios in pmol/g tissue were calculated, and results are expressed as a percentage of control values.

### 2.12 Statistical Analysis

Two-way ANOVA (group × treatment) followed by the *post hoc* Tukey b test was used. No statistical differences were detected between sham and naïve groups, and therefore, these animals were combined in a single control group. Three-way ANOVA (group × treatment × day) was used to analyze the acquisition phase of the MWM test. Statistical data are collected in Supplementary Table S1. SPSS v.24 software was used for all statistical analyses.

## 3 Results

### 3.1 Caf Treatment Restores Cognitive Deficits in Mice With GM-IVH

**FIGURE 1:**
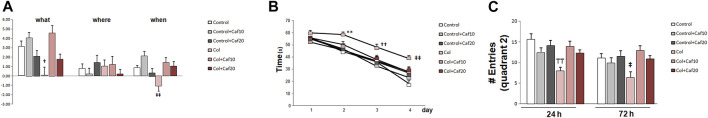
Caf treatment limits cognitive impairment after GM-IVH. **(A)** Col-treated mice were compromised in the NOD test for “what” and “when” paradigms. Caf treatment reverted this situation (“what” [F_(2,200)_ = 2.32 and *p* = 0.038; †*p* = 0.014 vs. Control + Caf10, Control + Caf20, and Col + Caf10]; “where” [F_(2,213)_ = 1.28 and *p* = 0.278]; “when” [F_(2,209)_ = 3.97 and *p* = 0.002; ‡‡*p* < 0.001 vs. Control, Control+ Caf10, Col + Caf10, and Col + Caf20]. Data are representative of 10–16 animals (Control n = 16, Control + Caf10 n = 15, Control + Caf20 n = 15, Col n = 10, Col + Caf10 n = 10, and Col + Caf20 n = 13). **(B)** Individual day assessment in the acquisition phase of the MWM revealed that GM-IVH impairs learning in the acquisition phase while Caf treatment limits this situation (day 1 [F_(5,305)_ = 1.72 and *p* = 0.128], day 2 [F_(5,307)_ = 0.315 and **p = 0.009 vs. rest of the groups], day 3 [F_(5,310)_ = 2.52 and ††*p* = 0.029 vs. Control, Control + Caf10, and Control+ Caf20], day 4 [F_(5,270)_ = 5.050 and ‡‡*p* < 0.01 vs. Control and Control+ Caf10]. Data are representative of 10–16 animals (Control n = 16, Control + Caf10 n = 16, control + Caf20 n = 16, Col n = 10, Col + Caf10 n = 10, and Col + Caf20 n = 12). **(C)** In the retention phase of the MWM, we analyzed the number of times that mice entered the quadrant where the platform used to be located (quadrant 2), and we observed that Col-injured mice entered a significantly lower number of times, while Caf reversed this situation during retention 1 (24 h) [F_(2,67)_ = 5.88 and *p* = 0.004; TT*p* = 0.003 vs. Control, Control + Caf10, Control + Caf20, and Col + Caf10]. A similar profile was observed in retention 2 (72 h) [F_(2,65)_ = 4.60 and *p* = 0.013; ‡*p* = 0.047 vs. Control + Caf10 and Control + Caf20]. Data are representative of 9–16 animals (Control n = 16, Control + Caf10 n = 15, Control + Caf20 n = 15, Col n = 9, Col + Caf10 n = 9, and Col + Caf20 n = 13).

We analyzed episodic memory in the NOD test and while we did not detect significant differences among groups for “where” paradigm, we observed a compromise for “what” and “when” paradigms in animals with GM-IVH. Nevertheless, Caf treatment (10 mg/Kg/day) significantly improved the performance in the “what” paradigm, while both doses of Caf (10 and 20 mg/Kg/day) restored the impairment observed for “when” paradigm (Figure 1A).

When analyzing learning and memory in the MWM, we did not detect a significant group × treatment × day effect in the acquisition phase [F_(61,196)_ = 1.18; *p* = 0.350]. However, the individual day assessment revealed a progressive compromise in mice with GM-IVH that was partially restored by Caf treatment. On day 2, both doses of Caf under study (10 and 20 mg/kg/day) significantly improved the performance in the MWM, and no differences were detected on the following days between control animals and animals with GM-IVH treated with Caf (Figure 1B). Memory impairment was also observed in animals with GM-IVH when the numbers of entrances in quadrant 2, where the platform was located along the acquisition phase, were compared. Animals treated with both doses of Caf treatment (10 and 20 mg/kg/day) reached control values 24 and 72 h after completing the acquisition phase, and Caf (10 mg/Kg/day) significantly improved the performance 24 h after completing the acquisition phase (Figure 1C).

The motor activity was not affected in any of the groups under study when we compared the total distance traveled in the open field, swimming speed in the MWM, time, and speed in the rotarod test (Table 1), suggesting that all observed behavioral outcomes are indeed due to learning and memory alterations and not due to motor alterations.

**TABLE 1:** Motor activity was not affected in any of the groups under study.

Treatment	Distance travelled (cm)	Time rotarod (s)	Speed rotarod (rpm)	Swimming velocity (cm/s)
Control	11,487.05 ± 865.02	14.06 ± 2.30	19.12 ± 2.02	27.53 ± 2.80
Control + Caf10	14,100.55 ± 840.67	12.73 ± 2.58	15.00 ± 1.72	22.79 ± 1.28
Control + Caf20	13,996.44 ± 1,688.70	13.66.1.80	20.13 ± 2.33	23.71 ± 1.03
Col	9,081.58 ± 1,085.24	10.37 ± 1.68	14.50 ± 2.40	25.57 ± 1.14
Col + Caf10	10,102.61 ± 1737.52	13.50 ± 2.70	17.80 ± 1.89	28.06 ± 2.95
Col + Caf20	11,489.95 ± 1,502.97	15.25 ± 1.54	20.07 ± 1.40	26.30 ± 2.92

Distance travelled in the motor activity test was not affected in any of the groups under study [F_(2,70)_ = 1.59; *p* = 0.211]. Similarly, time [F_(2,73)_ = 1.60; *p* = 0.209] and speed [F_(5,72)_ = 0.906; *p* = 0.482] in the rotarod test were similar in all groups under study. No differences were observed in the swimming velocity in the MWM test [F_(2,73)_ = 2.70; *p* = 0.074].

### 3.2 Brain Atrophy Is Reduced in GM-IVH Animals After Caf Treatment

**FIGURE 2:**
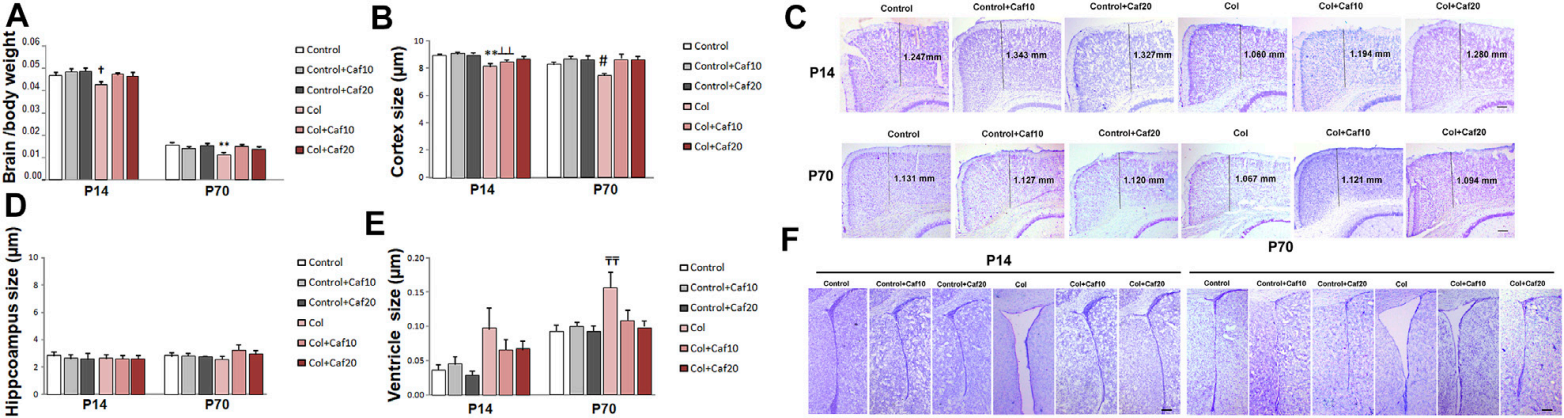
Brain atrophy was improved by Caf treatment. **(A)** Brain/body weight ratio was reduced in mice with GM-IVH, and this effect was limited by Caf treatment, at both 10 and 20 mg/kg/day in the short term [F_(2,87)_ = 4.24 and †*p* = 0.017 vs. Control, Control + Caf10 and Control + Caf20]. Data are representative of 9–21 mice (Control n = 16, Control + Caf10 n = 19, Control + Caf20 n = 18, Col n = 13, Col + Caf10 n = 13, and Col + Caf20 n = 17). Brain/body weight ratio was still reduced at P70 and Caf treatment restored this situation [F_(2,93)_ = 8.38 and **p < 0.01 vs. rest of the groups]. Data are representative of 10–21 mice (Control n = 21, Control + Caf10 n = 17, Control + Caf20 n = 19, Col n = 10, Col + Caf10 n = 15, and Col + Caf20 n = 17). **(B)** At P14, we detected a reduction of cortical size after Col lesions [F_(2,236)_ = 3.30 and *p* = 0.038, **p < 0.001 vs. rest of the groups, and ┴┴*p* < 0.001 vs. Control + Caf10], which was limited by Caf treatment, and a similar profile was observed at P70 [F_(2,187)_ = 3.32 and *p* = 0.038, #*p* = 0.007 vs. Control + Caf20, Col + Caf10, and Col + Caf20]. Data are representative of 6–8 mice (Control n = 8, Control + Caf10 n = 7, Control + Caf20 n = 6, Col n = 7, Col + Caf10 n = 7, and Col + Caf20 n = 8). **(C)** Representative cresyl violet staining images showing cortical size reduction after inducing GM-IVH and the restorative effect of Caf treatment at P14 and P70. Scale bar = 100 µm. **(D)** We did not observe any significant differences among groups when we compared the hippocampus size at P14 [F_(2,117)_ = 0.117 and *p* = 0.890]. Data are representative of 5–8 mice (Control n = 8, Control + Caf10 n = 7, Control + Caf20 n = 5, Col n = 7, Col + Caf10 n = 7, and Col + Caf20 n = 8). Differences did not reach statistical significance at P70 either [F_(2,83)_ = 0.779 and *p* = 0.453]. Data are representative of 4–7 mice (Control n = 6, Control + Caf10 n = 7, Control + Caf20 n = 4, Col n = 6, Col + Caf10 n = 4, and Col + Caf20 n = 7). **(E)** We detected ventricle enlargement after Col lesions, and Caf treatment limited this situation, reaching statistical significance in the long term: P14 [F_(2,107)_ = 0.198 and *p* = 0.5]. Data are representative of 6–8 mice (Control n = 8, Control + Caf10 n = 6, Control + Caf20 n = 6, Col n = 7, Col + Caf10 n = 7, and Col + Caf20 n = 8); P70 [F_(2,163)_ = 3.12 and *p* = 0.046; TT*p* = 0.005 vs. Control, Control + Caf10, Control + Caf20, and Col + Caf20]. Data are representative of 4–7 mice (Control n = 6, Control + Caf10 n = 7, Control + Caf20 n = 4, Col n = 6, Col + Caf10 n = 4, and Col + Caf20 n = 7). **(F)** Representative cresyl violet staining images showing ventricle enlargement after inducing GM-IVH and the restorative effect of Caf treatment at P14 and P70. Scale bar = 100 µm.

When we compared brain/body weight in all groups under study, we observed a significant reduction of this ratio in mice with GM-IVH in both the short (P14) and the long (P70) term. Our observations are in accordance with previous studies in a similar model (Jinnai et al., 2020), and also, patients with GM hemorrhage showed ventriculomegaly on the side of the hemorrhage with mild atrophy (Fusch et al., 1997). By P14, no differences were observed between control animals and those with GM-IVH treated with Caf. In the long term (P70), brain/body ratios were significantly improved by Caf (10 and 20 mg/Kg/day) when treated animals were compared with untreated GM-IVH mice (Figure 2A).

Further assessment of brain morphology revealed a compromise in animals with GM-IVH when we analyzed the cortical size. A significant reduction of the cortex size was observed, and Caf treatment limited this effect when animals were analyzed at P14. By P70, Caf treatment (10 and 20 mg/kg/day) significantly improved the cortical size when compared with those from untreated animals (Figures 2B,C). No significant differences among groups were observed when we compared the hippocampus size (Figure 2D). Nevertheless, brain atrophy was also supported by a significant ventricle enlargement detected in mice with GM-IVH both in the short (P14) and in the long term (P70), as previously described (Segado-Arenas et al., 2017). No differences were observed between control animals and GM-IVH mice treated with Caf in the short term (P14). Also, Caf 10 and 20 mg/kg/day significantly reduced ventricle enlargement at P70, when treated mice were compared with animals with GM-IVH, supporting the neuroprotective effect of the treatment (Figures 2E,F).

### 3.3 Caf Treatment Restores Neuron Density and Curvature

**FIGURE 3:**
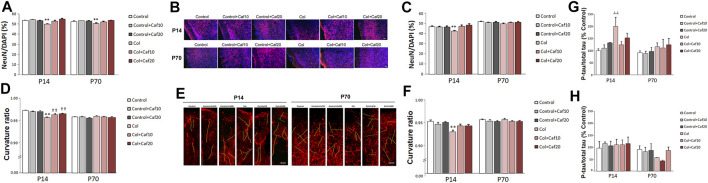
Caf reduces neuronal compromise after GM-IVH. **(A)** NeuN/DAPI ratio was significantly reduced in the cortex from Col-treated mice, and Caf treatment reverted this situation at P14 [F_(2,271)_ = 9.53 and *p* < 0.001; **p < 0.01 vs. rest of the groups]. Data are representative of 3–5 mice (Control n = 4, Control + Caf10 n = 5, Control + Caf20 n = 5, Col n = 3, Col + Caf10 n = 4, and Col + Caf20 n = 4). Similar differences were observed at P70 [F_(22,983)_ = 4.45 and *p* = 0.012; **p < 0.01 vs. rest of the groups]. Data are representative of 3–6 mice (Control n = 6, Control + Caf10 n = 5, Control + Caf20 n = 5, Col n = 4, Col + Caf10 n = 4, and Col + Caf20 n = 3). **(B)** Representative images of cortical NeuN (red) and DAPI (blue) staining showing that the reduced NeuN/DAPI ratio in GM-IVH animals improves in Caf-treated animals at P14 and P70. Scale bar = 50 µm. **(C)** NeuN/DAPI ratio was affected in the SVZ at P14 [F_(2,420)_ = 11.82 and *p* < 0.01; **p < 0.01 vs. rest of the groups]. Data are representative of 3–5 mice (Control n = 4, Control + Caf10 n = 5, Control + Caf20 n = 5, Col n = 3, Col + Caf10 n = 3, and Col + Caf20 n = 3). Differences were no longer detected at P70 [F_(2,559)_ = 0.351 and *p* = 0.704]. Data are representative of 3–6 mice (Control n = 6, Control + Caf10 n = 5, Control + Caf20 n = 5, Col n = 4, Col + Caf10 n = 4, and Col + Caf20 n = 3). **(D)** Cortical curvature ratio was significantly affected by Col lesions, and Caf treatment improved this situation at P14 [F_(24,048)_ = 16.79 and *p* < 0.01; **p < 0.01 vs. rest of the groups, ††*p* < 0.01 vs. Control, Control + Caf10, and Control + Caf20]. Data are representative of 5–6 mice (Control n = 5, Control + Caf10 n = 5, Control + Caf20 n = 5, Col n = 6, Col + Caf10 n = 6, and Col + Caf20 n = 5). No differences were observed in any of the groups under study when the curvature ratio was analyzed in the cortex at P70 [F_(23,645)_ = 2.65, *p* = 0.071]. Data are representative of 4–5 mice (Control n = 5, Control + Caf10 n = 5, Control + Caf20 n = 4, Col n = 5, Col + Caf10 n = 5, and Col + Caf20 n = 5). **(E)** Illustrative example of cortical SMI-312 staining at P14 and P70. Representative neurites are labeled in yellow to highlight neuronal curvature in all groups under study. Scale bar = 15 µm. **(F)** Curvature ratio was also significantly affected in the SVZ at P14 [F_(21,129)_ = 7.27 and *p* = 0.001; **p < 0.01 vs. rest of the groups]. Data are representative of 4–5 mice (Control n = 5, Control + Caf10 n = 5, Control + Caf20 n = 4, Col n = 5, Col + Caf10 n = 5, and Col + Caf20 n = 5). Neuron curvature ratio was no longer affected in the SVZ at P70 [F_(21,016)_ = 0.269 and *p* = 0.765]. Data are representative of 4–5 mice (Control n = 5, Control + Caf10 n = 5, Control + Caf20 n = 3, Col n = 5, Col + Caf10 n = 5, and Col + Caf20 n = 5). **(G)** Tau phosphorylation was significantly increased in the cortex from P14 mice after Col lesions, and Caf treatment improved this situation when compared with Control mice [F_(2,30)_ = 3.33 and *p* = 0.49; ┴┴*p* = 0.006 vs. Control and Control + Caf10]. Data are representative of 5–7 animals (Control n = 7, Control + Caf10 n = 5, Control + Caf20 n = 5, Col n = 7, Col + Caf10 n = 6, and Col + Caf20 n = 6). Differences were no longer detected in the long term [F_(2,26)_ = 0.016 and *p* = 0.984]. Data are representative of 4–7 animals (Control n = 5, Control + Caf10 n = 5, Control + Caf20 n = 4, Col n = 7, Col + Caf10 n = 6, and Col + Caf20 n = 5). **(H)** Tau phosphorylation was not significantly affected in the striatum from P14 mice [F_(2,27)_ = 0.031 and *p* = 0.970]. Data are representative of 5–6 animals (Control n = 5, Control + Caf10 n = 5, Control + Caf20 n = 6, Col n = 6, Col + Caf10 n = 5, and Col + Caf20 n = 6). No differences were observed among groups at P70 [F_(2,25)_ = 0.768 and *p* = 0.474]. Data are representative of 5–6 animals (Control n = 5, Control + Caf10 n = 5, Control + Caf20 n = 5, Col n = 5, Col + Caf10 n = 5, and Col + Caf20 n = 6).

We also characterized the neuron density and neuritic curvature as a marker of neuron wellbeing, as previously described (Stern et al., 2004; Meyer-Luehmann et al., 2008). Mature neuron density was quantified by measuring the NeuN/DAPI ratio. We observed that this ratio was significantly compromised in the cortex both in the short (P14) and the long (P70) term, after inducing a GM-IVH, while Caf treatment (10 and 20 mg/kg/day) significantly improved this situation at both time points, reaching control values for both 10 and 20 mg/kg/day (Figures 3A,B) and suggesting a role in protecting neuronal integrity after the lesions. When we analyzed the SVZ, we observed a reduced NeuN/DAPI ratio in animals with a lesion that was significantly improved in the short term (P14) after 10 and 20 mg/kg/day Caf was administered. Differences were not statistically significant in the long term (P70) (Figure 3C).

We also analyzed neuron wellbeing by measuring the neurite curvature since increased curvature ratios are observed in neurons under other insults (Meyer-Luehmann et al., 2008; Infante-Garcia et al., 2017b). At P14, the neurite curvature was severely compromised in the cortex from animals with GM-IVH when compared with the rest of the groups. A significant improvement was observed after Caf treatment at both doses under study (10 and 20 mg/kg/day) when compared with untreated mice with GM-IVH, although they did not reach control values (Figures 3D,E). Differences in the neurite curvature were no longer detected among groups when the cortex was analyzed in the long term (P70) (Figures 3D,E). At P14, neurite curvature was significantly affected in the SVZ from animals lesioned with Col, and Caf (10 and 20 mg/kg/day) completely reversed this limitation (Figure 3F). On the other hand, as observed in the cortex, differences among groups were no longer observed when the neurite curvature was analyzed in the long term (P70) in the SVZ.

### 3.4 Caf Treatment Limits Cortical Tau Hyperphosphorylation

When we analyzed the cortex from GM-IVH, we observed an increase in [pS199] tau phosphorylation by 14 days of age. Abnormal tau phosphorylation is observed in different neuropathological situations as a marker of neuronal damage, and interestingly, even early tau alterations might significantly worsen cognitive function (Hochgräfe et al., 2013). The reduction observed in the phospho tau/total tau ratio after the Caf treatment was not statistically significant when compared with untreated animals, although differences were no longer detected when Caf-treated mice were compared with control animals (Figure 3G). By P70, tau hyperphosphorylation was no longer observed in the cortex from animals with a lesion, and no differences were detected among groups (Figure 3G). When we analyzed the striatum, we did not observe any significant differences in tau phosphorylation in the short (P14) or the long (P70) term (Figure 3H).

### 3.5 Neurogenesis Impairment Is Improved by Caf Treatment After Inducing GM-IVH

**FIGURE 4:**
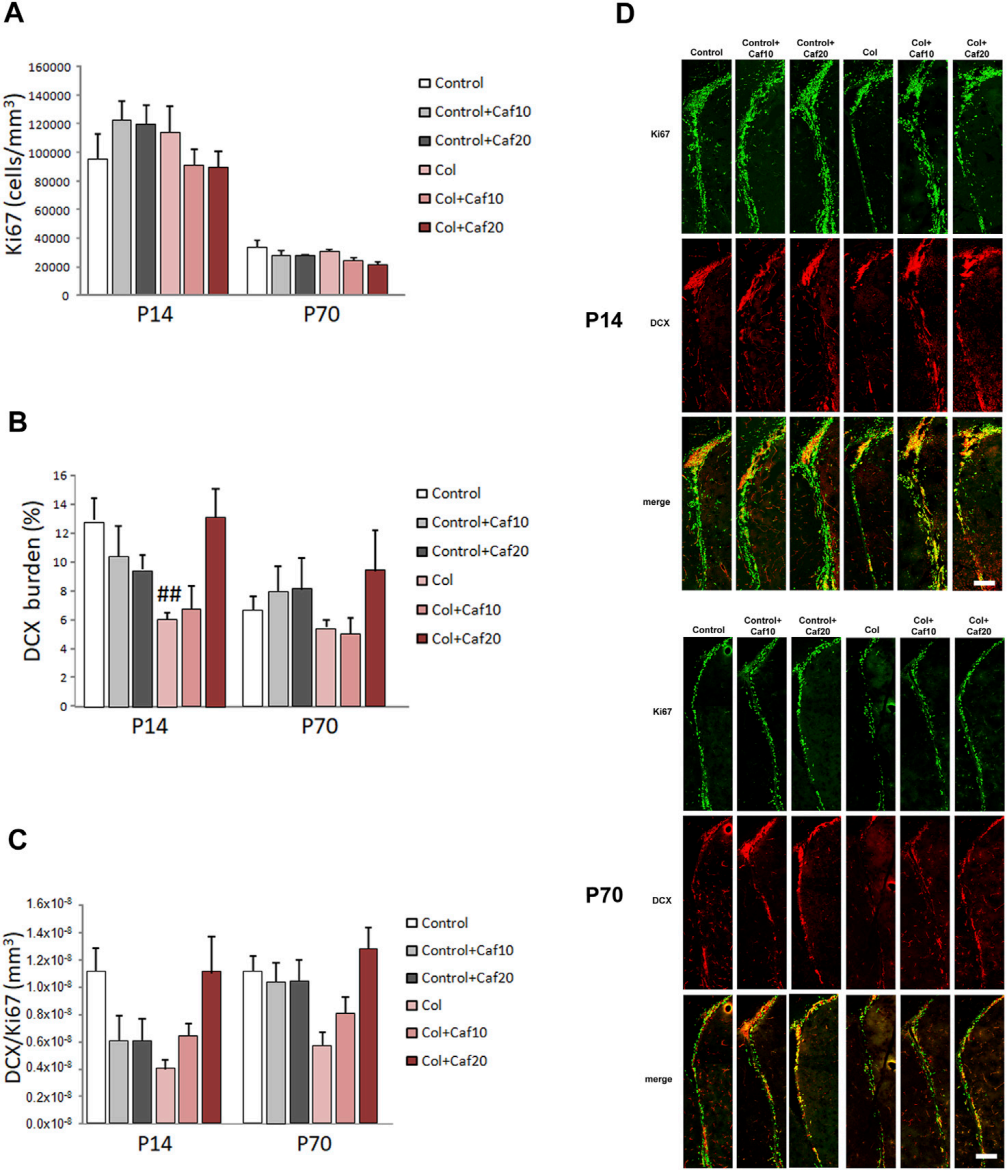
Caf treatment improves neurogenesis in the SVZ after GM-IVH. **(A)** Proliferating cells were not significantly affected in the SVZ when we analyzed the density of Ki67^+^ cells in the short (P14) [F_(2,94)_ = 1.74 and *p* = 0.181] or the long (P70) [F_(2,85)_ = 0.023 and *p* = 0.977] term. **(B)** DCX burden was significantly reduced in the SVZ after GM-IVH induction. The highest dose of Col (20 mg/kg/day) counterbalanced this situation at P14 [F_(2,95)_ = 5.86 and *p* = 0.003; ##*p* = 0.004 vs. Control and col + Caf20], and a similar profile was observed at P70, although differences were not statistically significant [F_(2,83)_ = 0.694 and *p* = 0.503]. **(C)** DCX area/Ki67^+^ cell ratio was also reduced after Col lesions, although differences were not statistically significant at P14 [F_(2,96)_ = 0.00 and *p* = 1.00] or P70 [F_(2,84)_ = 0.00 and *p* = 1.00]. Data are representative of 6–7 animals (P14: Control n = 7, Control + Caf10 n = 6, Control + Caf20 n = 6, Col n = 7, Col + Caf10 n = 6, and Col + Caf20 n = 6. P70: Control n = 7, Control + Caf10 n = 6, Control + Caf20 n = 6, Col n = 7, Col + Caf10 n = 6, and Col + Caf20 n = 6). **(D)** Illustrative example of Ki67 (green) and DCX (red) immunostaining in the SVZ from mice with GM-IHV treated with Caf at P14 and P70. Scale bar = 125 µm.

We analyzed proliferation and neurogenesis (by Ki67 and DCX immunostaining respectively) in the SVZ, a major neurogenic niche in the mouse. We detected an overall reduction in the number of Ki67^+^ cells and DCX^+^ area from P14 to P70, as previously described in other models (Hierro-Bujalance et al., 2020a), since both processes are reduced with age. We did not observe significant differences in the number of proliferating cells after GM-IVH lesions or after Caf treatment in the short (P14) or in the long (P70) term (Figures 4A,D). However, we observed a compromise in neurogenesis, and the DCX burden was severely reduced in the SVZ at P14 after Col lesions, probably as a consequence of the damage in the area induced by Col administration, as previously described (Segado-Arenas et al., 2017). Nevertheless, Caf treatment at the highest dose (20 mg/kg/day) improved this situation in the short term (P14) reaching control values, suggesting that beneficial effects mediated by Caf might be related to its capacity to preserve brain neurogenesis. While a similar profile was observed in the long term (P70), differences did not reach statistical significance (Figures 4B,D). We also observed a compromise when we analyzed DCX area/Ki67^+^ cells in the SVZ, although differences were not statistically significant (Figures 4C,D).

### 3.6 Bleeding Is Reduced by Caf Treatment After GM-IVH

**FIGURE 5:**
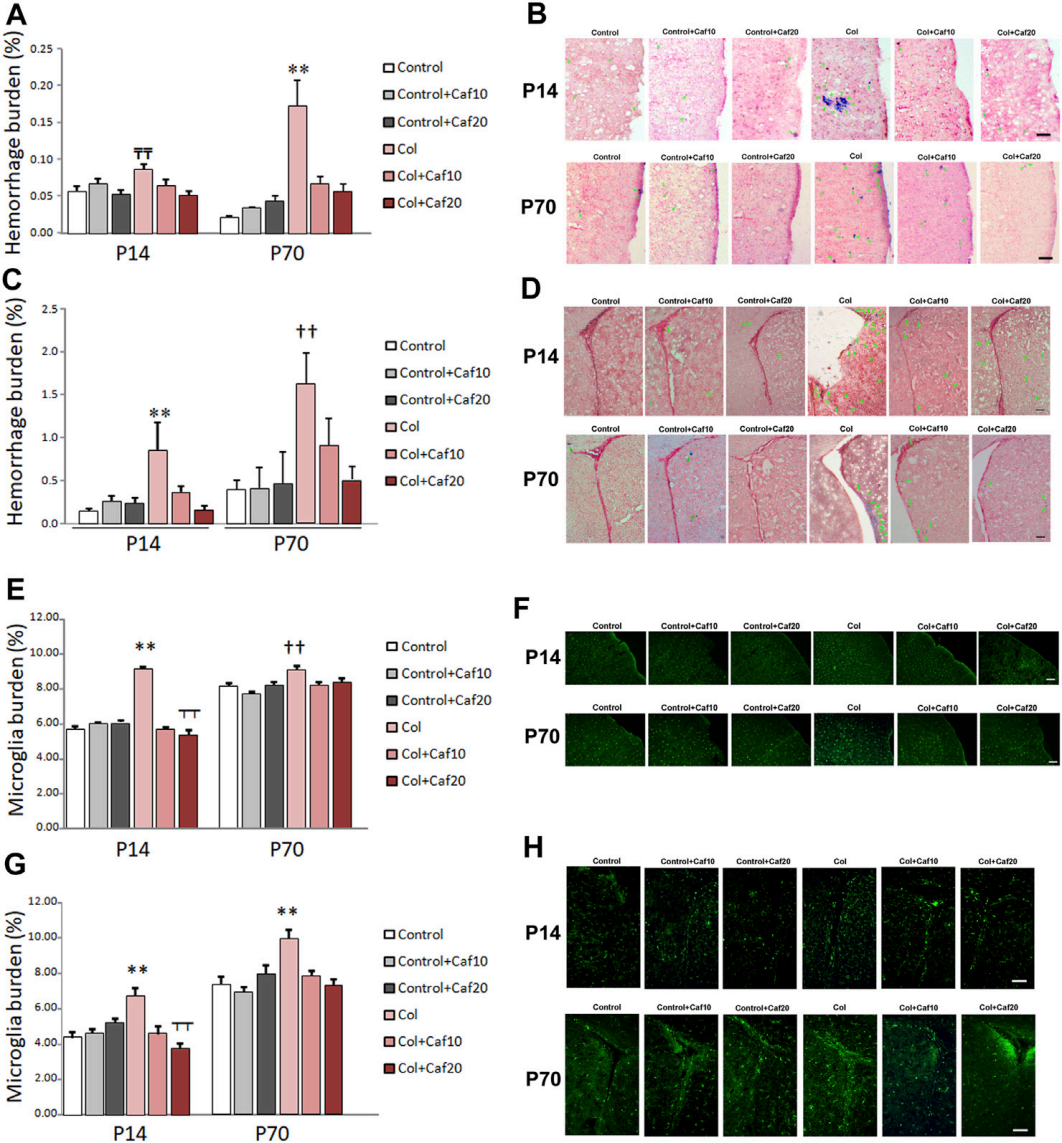
Caf reduces bleeding and inflammation in the brain from animals with GM-IVH. **(A)** Hemorrhage burden was increased in the cortex from animals with Col lesions, and Caf treatment limited this situation in the short term (P14) [F_(2,150)_ = 4.27 and *p* = 0.016; TT*p* = 0.001 vs. Control, Control + Caf20, and Col + Caf20] and completely reversed it in the long term (P70) [F_(2,153)_ = 12.02 and *p* < 0.001; **p = 0.001 vs. rest of the groups]). Data are representative of 4–6 animals (P14: Control n = 6, Control + Caf10 n = 5, Control + Caf20 n = 5, Col n = 6, Col + Caf10 n = 6, and Col + Caf20 n = 6. P70: Control n = 5, Control + Caf10 n = 5, Control + Caf20 n = 4, Col n = 6, Col + Caf10 n = 4, and Col + Caf20 n = 5). **(B)** Illustrative example of Prussian blue staining in the cortex from animals with GM-IVH lesions and after treatment with Caf. Green arrows point at hemorrhages. Scale bar = 100 µm. **(C)** Increased hemorrhage burden in the SVZ was also reduced after Caf treatment at P14 [F_(2,73)_ = 5.93 and *p* = 0.004; **p = 0.003 vs. rest of the groups] and P70 [F_(2,71)_ = 4.74 and *p* = 0.012; ††*p* = 0.010 vs. Control, Control + Caf10, Control + Caf20, and Col + Caf20]. Data are representative of 4–6 animals (P14: Control n = 6, Control + Caf10 n = 5, Control + Caf20 n = 5, Col n = 6, Col + Caf10 n = 5, and Col + Caf20 n = 5. P70: Control n = 5, Control + Caf10 n = 5, Control + Caf20 n = 4, Col n = 6, Col + Caf10 n = 4, and Col + Caf20 n = 5). **(D)** Illustrative example of Prussian blue staining in the SVZ from animals with GM-IVH lesions and after treatment with Caf. Green arrows point at hemorrhages. Scale bar = 100 µm. **(E)** Microglial burden was significantly increased after Col lesions, and Caf treatment reverted this situation at P14 in the cortex [F_(21,814)_ = 87.42 and *p* < 0.001; **p < 0.01 vs. rest of the groups and ┬┬*p* < 0.01 vs. Control + Caf20]. A similar profile was observed at P70 in the cortex [F_(22,914)_ = 6.22 and *p* = 0.002; ††*p* < 0.01 vs. Control, Control + Caf10, Col + Caf10, and Col + Caf20]. Data are representative of 3–5 animals (P14: Control n = 3, Control + Caf10 n = 5, Control + Caf20 n = 5, Col n = 3, Col + Caf10 n = 3, and Col + Caf20 n = 4. P70: Control n = 5, Control + Caf10 n = 6, Control + Caf20 n = 5, Col n = 4, Col + Caf10 n = 4, and Col + Caf20 n = 3). **(F)** Illustrative images of cortical Iba-1 staining in the cortex from all groups under study at P14 and P70. Scale bar = 100 µm. **(G)** GM-IVH also increased microglial burden in the SVZ, and Caf reversed this situation at both P14 [F_(2,335)_ = 12.11 and *p* < 0.001; **p < 0.01 vs. rest of the groups and ┬┬*p* < 0.01 vs. Control + Caf20] and P70 [F_(2,547)_ = 7.14 and *p* = 0.001; **p < 0.01 vs. rest of the groups]. **(H)** Illustrative images of cortical Iba-1 staining in the SVZ from all groups under study at P14 and P70. Scale bar = 100 µm.

As previously shown, induction of GM-IVH results in an overspread increase in small vessel bleeding in the brain (Segado-Arenas et al., 2017; Hierro-Bujalance et al., 2020b), supporting the idea that vascular damage is not only limited to the site of the injection but also showing alterations in more distant regions. In our hands, Caf reduced vascular damage analyzed by the presence of hemorrhages in the brain. When we analyzed the cortex, we observed that hemorrhage burden was significantly increased at P14, while no differences were observed when Caf-treated animals were compared with control mice. Cortical hemorrhage burden was also significantly increased in the long term (P70) in animals with GM-IVH, whereas Caf treatment effectively reduced the presence of cortical hemorrhages when administered at 10 and 20 mg/kg/day (Figures 5A,B). As expected, the SVZ was the most severely affected area due to its proximity to the ventricle, and Caf treatment successfully reduced hemorrhage burden at P14. A similar profile was observed at P70, although statistical differences with GM-IVH animals were only observed at the highest dose of Caf under study (20 mg/kg/day) (Supplementary Table S2) (Figures 5C,D).

### 3.7 Caf Treatment Reduces Inflammation in Animals With GM-IVH

Microglia burden, analyzed by Iba-1 staining, was significantly higher in the cortex from animals with GM-IVH, at both P14 and P70, and Caf (10 and 20 mg/kg/day) reduced cortical microglial burden (Figures 5E,F). Similarly, microglial burden was also increased in the SVZ from animals with Col lesions in the short (P14) and the long (P70) term, and both doses of Caf effectively reduced the presence of activated microglia (Figures 5G,H). Our observations are in line with previous studies showing the Caf anti-inflammatory activity (Yang et al., 2022).

## 4 Discussion

GM-IVH is one of the most relevant brain complications of the PT (Radic et al., 2015b; Valdez Sandoval et al., 2019), responsible for severe disabilities in the majority of these patients (Segado-Arenas et al., 2017), including cognitive and motor impairments (Morita et al., 2015; He et al., 2019), developmental delay (Bolisetty et al., 2014; Holwerda et al., 2016; Matijevic et al., 2019), or cerebral palsy (Bolisetty et al., 2014; da Silva et al., 2018). Even though the consequences are devastating GM-IVH has no successful treatment, and patients are in a tremendous need of new therapeutic opportunities.

Caf is commonly used in the clinic to treat the apnea of prematurity (Abdel-Hady et al., 2015), and previous studies have reported the beneficial effects of Caf treatment not only at the pulmonary level but also on the CNS. Caf treatment is not associated with improved survival rates without disabilities in VLBWI at 5 years (Schmidt et al., 2012). However, developmental coordination disorders are reduced by this age (Doyle et al., 2014). Also, Caf has been shown to have positive effects at an earlier age (18–21 months) with lower mean costs for these patients and better survival rates without neurodevelopmental disabilities, including reduced incidence of cerebral palsy and cognitive delay (Schmidt et al., 2007; Dukhovny et al., 2011). Moreover, long-term analysis reveals that by 11 years of age, kids treated with Caf for apnea of prematurity had better visuomotor, visuoperceptual, and visuospatial abilities or fine motor coordination (Maitre et al., 2015; Schmidt et al., 2017; Mürner-Lavanchy et al., 2018). In line with these observations, early Caf treatment to PT improves white matter microstructural development (Doyle et al., 2010; Liu et al., 2020). These data support that Caf has a direct neuroprotective effect apart from improving respiratory function (Yang et al., 2021). Since Caf is used to treat respiratory dysfunction, most of the studies focus on secondary outcomes, and the work specifically addressing the role of Caf on GM-IVH is limited in patients and animal models.

We have analyzed the direct effect of Caf in brain complications and cognitive function in a murine model of GM-IVH, generated by intracerebroventricular administration of 0.3 IU of Col. Previous studies have established this murine model of GM-IVH based on Col administration to P7 rodents (Lekic et al., 2015), and later studies have also used this approach (Segado-Arenas et al., 2017; Tang et al., 2017; Li et al., 2018; Scheuer et al., 2018; Almeida et al., 2019; Hierro-Bujalance et al., 2020b). Since P7 mice might be considered in the limit of prematurity (Semple et al., 2013), other approaches have also used younger mice (Alexander et al., 2014; Ko et al., 2018; Del Pozo et al., 2022) that might resemble a more severe aspect of prematurity, and therefore, it is also important to bear in mind that the actual outcomes might differ depending on the actual postnatal day in which the lesions are induced.

Earlier investigations have already shown that neonatal mice and PT express receptors for Caf (adenosine receptors 1 and 2) in the brain (Li et al., 2019), supporting the direct effect of Caf on the CNS (Li et al., 2019). We observed that Caf treatment, at both 10 and 20 mg/kg/day, had a neuroprotective effect in the short (P14) and the long (P70) term. The brain/body weight ratio was significantly reduced after Col administration, and Caf treatment restored control values in animals with GM-IVH. Direct brain examination revealed an overall compromise that preferentially affected the cortex at P14 and P70. Caf treatment successfully restored cortical atrophy and reduced ventricle enlargement, classically observed in GM-IVH patients (Goeral et al., 2021; Szentimrey et al., 2022), as well as in this (Segado-Arenas et al., 2017; Hierro-Bujalance et al., 2020b), and in other models resembling brain complications of the PT (Alexander et al., 2013; Di Martino et al., 2020).

Neuron wellbeing was further assessed by analyzing neurite curvature, as previously performed in other pathologies (Serrano-Pozo et al., 2010; Mizutani et al., 2021). Neurite curvature was significantly affected in the cortex and the SVZ at P14 in mice with GM-IVH, while both doses of Caf successfully limited this effect. We also observed an increase in tau phosphorylation in the cortex as it occurs in neurodegenerative disorders, indicative of alterations in the axonal microtubule assembly (Johnson and Stoothoff, 2004; Segado-Arenas et al., 2017; Trushina et al., 2019), and Caf treatment reduced tau hyperphosphorylation, in line with previous observations (Laurent et al., 2016; Zhao et al., 2017). Likewise, neuron population assessed by the NeuN/DAPI ratio was reduced after GM-IVH in the cortex and the SVZ from mice with GM-IVH, and Caf treatment counterbalanced this situation. These data support the capacity of Caf to limit neuronal loss, in line with previous studies showing that Caf may promote neuron survival after an hypoxic situation (Li et al., 2019; Soontarapornchai et al., 2021) or other insults (Stazi et al., 2021; Xie et al., 2021).

Since GM-IVH directly affects the SVZ, a main neurogenic niche in mice, we also analyzed cell proliferation and neurogenesis after Caf treatment. Previous studies have revealed a compromise in neurogenesis in this model (Segado-Arenas et al., 2017), and we also observed a reduction in DCX labeling in the SVZ. Interestingly, Caf treatment at the highest dose (20 mg/kg/day) successfully restored neurogenesis impairment, suggesting that Caf effects might not be circumscribed to limiting neuronal loss, but it may also promote neurogenesis. Whereas some studies have shown that Caf may compromise proliferation of human hippocampal progenitor cells (Houghton et al., 2020), neurogenesis seems to improve after Caf treatment in different animal models (Mao et al., 2020; Stazi et al., 2021).

We further analyzed the effect of Caf on small vessel bleeding observed after the induction of GM-IVH (Segado-Arenas et al., 2017; Hierro-Bujalance et al., 2020b). As expected, Col lesions increased the presence of hemorrhages in the cortex and more severely in the areas surrounding the injection site, as the SVZ. Previous studies in patients have shown that early Caf administration improves hemodynamics (Katheria et al., 2015), and given the fragility of the preterm vasculature, this may account for the protective effects observed. While both doses of Caf reduced hemorrhage burden, Caf 20 mg/kg/day had a more robust effect. At this point, it should be taken into consideration that the doses used in this study (10 and 20 mg/kg/day) are in the range of previous studies using Caf in other models showing beneficial effects (Kaindl et al., 2008; Fan et al., 2011), but high-dose Caf treatment might have a negative impact on brain development and associated complications and therefore, Caf effects might entirely depend on the actual dose and pathologies under study (McPherson et al., 2015; Vesoulis et al., 2016; Saroha and Patel, 2020; Soontarapornchai et al., 2021). Importantly, Caf might not only have a positive effect in limiting the pathological complications associated, but it may also reduce the incidence of GM-IVH itself, when administered early to patients at risk (Borszewska-Kornacka et al., 2017; Helwich et al., 2017).

Antioxidant and anti-inflammatory properties of Caf have been largely addressed in different animal models, including neonatal hypoxia-ischemia or neonatal hyperoxia models (Endesfelder et al., 2017b; Di Martino et al., 2020), downregulating pro-inflammatory cytokines or limiting the presence of amoeboid microglia. Caf also suppresses pro-inflammatory mediators and their regulatory genes after lipopolysaccharide insult to microglial cells (Kang et al., 2012). However, to the best of our knowledge, no previous studies have addressed the anti-inflammatory effects of Caf after GM-IVH. Our study shows a significant increase in microglial burden in the cortex, and more importantly in the SVZ, after lesions, in both the short and the long term, whereas Caf treatment effectively counterbalances this inflammatory phenotype. In line with this, previous studies have reported that activation of adenosine A2a receptors might modulate microglial activation in animal models of perinatal brain injury (Colella et al., 2018). Following this idea, it is feasible that reduction of the inflammatory process might contribute to observed neuroprotection after Caf treatment, as previously described in hypoxic-ischemic damage in neonatal rats where Caf, through A2a receptors, inhibits the activation of NLRP3 inflammasome, reduces microglial activation, and regulates the release of inflammatory factors (Yang et al., 2022). However, we cannot exclude that other mechanisms may also contribute to the observed positive effects of Caf on neuron stability and wellbeing and previous studies in other models of neonatal insults have shown that Caf successfully reduces apoptosis markers (Endesfelder et al., 2017a; Soontarapornchai et al., 2021). On the other hand, other studies have pointed out that Caf neuroprotective effects might also be mediated by the regulation of autophagy in different models of neurodegeneration (Moon et al., 2014; Luan et al., 2018).

Cognitive impairments were observed in mice after GM-IVH. Although it might be possible that hippocampus is affected by Col administration, similar outcomes have been reported in other models of GM-IVH (Li et al., 2018), and our results are also in accordance with cognitive alterations observed in patients (Vohr, 2022). Episodic and spatial memory were improved after Caf treatment, and although no previous studies have addressed the role of Caf on cognitive impairment associated with GM-IVH lesions, our results are in line with other observations showing the beneficial effects of Caf and other methylxanthines after hypoxic-ischemic insults in newborn animals (Kumral et al., 2010; Potter et al., 2018). Although we cannot point toward a specific pathological feature responsible for cognitive impairment in our mouse model, it is feasible that the combination of all alterations might result in learning and memory disabilities observed since previous studies have shown independent beneficial effects for Caf that result in cognitive improvement after different insults (Alexander et al., 2013; Zhao et al., 2017; Potter et al., 2018).

Caf is a commonly used drug to treat the apnea of prematurity, and although it may also improve brain-associated complications to GM-IVH (Helwich et al., 2017), no previous study has addressed the role of Caf in brain pathology and cognitive impairment at this level. We showed that Caf counterbalances brain atrophy and neuronal damage while limiting small vessel bleeding and inflammation, ultimately ameliorating cognitive impairment and supporting a feasible role for Caf to reduce complications associated with GM-IVH of the VLBWI.

## Data Availability

The raw data supporting the conclusion of this article will be made available by the authors, without undue reservation.
