# Supplementary figures and images for: Caffeine Restores Neuronal Damage and Inflammatory Response in a Model of Intraventricular Hemorrhage of the Preterm Newborn
Source: Front Cell Dev Biol. 2022 Aug 12;10:908045. doi: 10.3389/fcell.2022.908045 (PMC9411947; doi:10.3389/fcell.2022.908045)

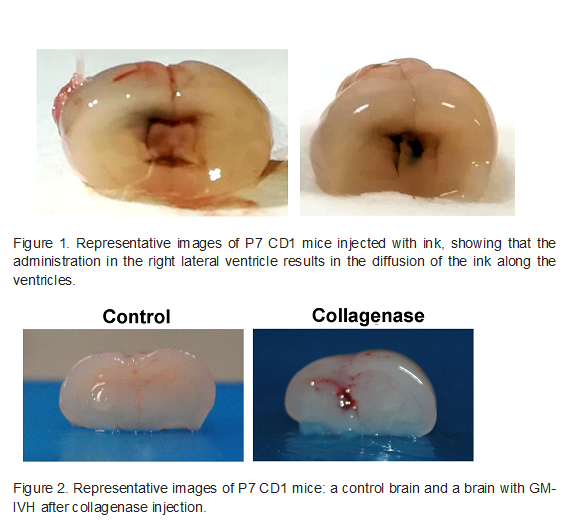

Supplement: Supplementary file 1 [file Image1.TIF]
