# Supplementary material for: Caffeine Restores Neuronal Damage and Inflammatory Response in a Model of Intraventricular Hemorrhage of the Preterm Newborn
Source: Front Cell Dev Biol. 2022 Aug 12;10:908045. doi: 10.3389/fcell.2022.908045 (PMC9411947; doi:10.3389/fcell.2022.908045)
Supplement: Supplementary file 2 [file DataSheet1.pdf]

Supplementary Table 1. Statistical data after analyzing the role of Caf in GM-IVH in the short (P14) and the long (P70) term.

Study	Short term (p14)	Long term (P70)
Distance travelled in the open field		[$F_{(2,70)}$=1.59, p=0.211]
Time in rotarod		[$F_{(2,73)}$=1.60, p=0.209]
Maximum speed in rotarod		[$F_{(5,72)}$=0.906, p=0.482]
Swimming velocity in the MWM		[$F_{(2,73)}$=2.70, p=0.074]
What paradigm in the NOD test		[$F_{(5,200)}$=2.32, p=0.038; †p=0.014 vs. Control+Caf10, Control+Caf20 and Col+Caf10]
Where paradigm in the NOD test		[$F_{(5,213)}$=1.28, p=0.278]
When paradigm in the NOD test		[$F_{(2,209)}$=3.97, p=0.002; ‡‡p<0.001 vs. Control, Control+Caf10, Col+Caf10 and Col+Caf20]
Time along acquisition phase of the MWM		day 1 [$F_{(5,305)}$=1.72, p=0.128]<br>day 2 [$F_{(5,307)}$=0.315, **p=0.009 vs. rest of the groups<br>day 3 [$F_{(5,310)}$=2.52, ††‡p=0.029 vs. Control, Control+Caf10 and Control+Caf20]<br>day 4 [$F_{(5,270)}$=5.050, ‡‡p<0.01 vs. Control and Control+Caf10]
Number of entrances in quadrant 2 in the retention 1 of the MWM		[$F_{(2,67)}$=5.88, p=0.004; ŦŦp=0.003 vs. Control, Control+Caf10, Control+Caf20, Col+Caf10]
Number of entrances in quadrant 2 in the retention 2 of the MWM		[$F_{(2,65)}$=4.60, p=0.013; ‡p=0.047 vs. Control+Caf10 and Control+Caf20]
Brain/body weight ratio	[$F_{(2,87)}$= 4.24, †p=0.017 vs. Control, Control+Caf10 and Control+Caf20]	[$F_{(2,93)}$= 8.38, **p<0.01 vs. rest of the groups]
Cortex size	$F_{(5,236)}$=3.30, p=0.038, **p<0.001 vs. rest of the groups, ┴┴p<0.001 vs. Control+caf10]	[$F_{(2,187)}$=3.32, p=0.038, #p=0.007 vs. Control+Caf20, Col+Caf10 and Col+Caf20]
Hippocampus size	[$F_{(2,117)}$=0.117, p=0.890]	[$F_{(2,83)}$=0.779, p=0.453]
Ventricle size	[$F_{(2,107)}$=0.198, p=0.5]	[$F_{(2,163)}$=3.12, p=0.046; ŦŦp=0.005 vs. Control, Control+Caf10,

		Control+Caf20, Col+Caf20]
Cortex NeuN/DAPI ratio	[$F_{(2,271)}$=9.53, p<0.001; **p<0.01 vs. rest of the groups]~~.~~	[$F_{(2,2983)}$=4.45, p=0.012; **p<0.01 vs. rest of the groups]
SVZ NeuN/DAPI ratio	P14 [$F_{(2,420)}$=11.82, p<0.01; **p<0.01 vs. rest of the groups]	[$F_{(2,559)}$=0.351, p=0.704]
Cortex neurite curvature ratio	[$F_{(2,4048)}$=16.79, p<0.01; **p<0.01 vs. rest of the groups, ††p<0.01 vs. Control, Control+Caf10 and Control+Caf20]	[$F_{(2,3645)}$=2.65, p=0.071]
SVZ neurite curvature ratio	$F_{(2,1129)}$=7.27, p=0.001; **p<0.01 vs. rest of the groups]	[$F_{(2,1016)}$=0.269, p=0.765]
Cortex P-tau/ total tau ratio	[$F_{(2,30)}$=3.33, p=0.49; ⊥⊥p=0.006 vs. Control and Control+Caf10]	[$F_{(2,26)}$=0.016, p=0.984]
Striatum P-tau/total tau ratio	[$F_{(2,27)}$=0.031, p=0.970]	[$F_{(2,25)}$=0.768, p=0.474]
Density of Ki67^{+} cells	[$F_{(2,94)}$=1.74 p=0.181]	[$F_{(2,85)}$=0.023, p=0.977]
DCX burden	[$F_{(2,95)}$=5.86, p=0.003; ##p=0.004 vs. Control and C~~e~~ol+Caf20]	[$F_{(2,83)}$=0.694, p=0.503]
DCX/ Ki67^{+} ratio	[$F_{(2,96)}$=0.00, p=1.00]	[$F_{(2,84)}$=0.00, p=1.00]
Cortex hemorrhage burden	[$F_{(2,150)}$=4.27, p=.016; ŦŦ p=0.001 vs. Control, Control+Caf20 and Col+Caf20]	[$F_{(2,153)}$=12.02, p<0.001, **p=0.001 vs. rest of the groups]
SVZ hemorrhage burden	[$F_{(2,73)}$=5.93, p=0.004, **p=0.003 vs. rest of the groups]	[$F_{(2,71)}$=4.74, p=0.012, ††p=0.010 vs. Control, Control+Caf10, Control+Caf20 and Col+Caf20]
Cortex microglia burden	[$F_{(2,1814)}$=87.42, p<0.001, **p<0.01 vs. rest of the groups, ┬┬p<0.01 vs. Control+Caf20]	[$F_{(2,2914)}$=6.22, p=0.002; ††p<0.01 vs. Control, Control+Caf10, Col+Caf10 and Col+Caf20]
SVZ microglia burden	$F_{(2,335)}$=12.11, p<0.001; **p<0.01 vs. rest of the groups, ┬┬p<0.01 vs. Control+Caf20]	[$F_{(2,547)}$=7.14, p=0.001; **p<0.01 vs. rest of the groups]

Supplementary Table 2. Hemorrhage burden are limited by Caf treatment in the cortex and SVZ both in the short (P14) and the Long (P70) term.

Supplementary Table 2. Hemorrhage burden was ameliorated by Caf treatement in the cortex and the hippocampus in animilas with GM-IVH.

	**Hemorrhage burden (% area affected)**			
	**Cortex**		**SVZ**	
**Group**	**P14**	**P70**	**P14**	**P70**
**Control**	0.057±0.006	0.022±0.003	0.138±0.035	0.446±0.130
**Control+Caf10**	0.066±0.006	0.036±0.003	0.279±0.050	0.451±0.231
**Control+Caf20**	0.054±0.005	0.046±0.006	0.252±0.049	0.506±0.363
**Col**	0.084±0.007ŦŦ	0.173±0.035**	0.841±0.307**	1.634±0.362††
**Col+Caf10**	0.047±0.006	0.070±0.009	0.359±0.067	0.959±0.316
**Col+Caf20**	0.061±0.03	0.055±0.009	0.162±0.054	0.524±0.120

Hemorrhage burden was increased in the cortex from animals with Col lesions. Caf treatment limited this situation in the short [$F_{(2,150)}$=4.27, p=.016; ŦŦp=0.001 vs. Control, Control+Caf20 and Col+Caf20]. In the long term, Caf treatment completely counterbalanced increased cortical hemorrhage burden [$F_{(2,153)}$=12.02, p<0.001, **p=0.001 vs. rest of the groups]). Hemorrhage burden was also improved in the SVZ after Caf treatment at P14 [$F_{(2,73)}$=5.93, p=0.004, **p=0.003 vs. rest of the groups]. By P70 Caf at the highest dose (20 mg/Kg/day) successfully reduced hemorrhage burden in the SVZ [$F_{(2,71)}$=4.74, p=0.012, ††p=0.010 vs. Control, Control+Caf10, Control+Caf20 and Col+Caf20].
